# Enhancing soft robots with chemical shielding for harsh corrosive liquid environments

**DOI:** 10.1039/d5mh01593f

**Published:** 2025-12-10

**Authors:** Haitao Qing, Sravanthi Vallabhuneni, Yinding Chi, Mohammad Javad Zarei, Pouya Sharbati, Haoze Sun, Jie Yin, Arun Kumar Kota

**Affiliations:** a Department of Mechanical and Aerospace Engineering, North Carolina State University Raleigh NC 27695 USA jyin8@ncsu.edu akota2@ncsu.edu

## Abstract

Soft robots offer safe interactions and adaptability for underwater applications such as environmental monitoring. However, their operation in corrosive liquid environments remains a challenge due to the degradation of elastomeric components upon exposure to acids, bases, and organic solvents. Here, a universal chemical shielding strategy is introduced for elastomer-based soft robots using a spray-coated superomniphobic skin composed of fluorinated silica nanoparticles. The coating exhibits high contact angles (>150°) and low roll-off angles (<10°) for liquids spanning a wide range of surface tensions, preventing wetting and protecting against strong acids and organic solvents. The strategy is applied to representative actuators made of silicone rubber, liquid crystal elastomers, and magnetic elastomer composites, actuated by pneumatic pressure, infrared light, and magnetic fields, respectively. These coated soft robots exhibit robust swimming, crawling, shape morphing, and manipulation without degradation under harsh chemical environments consisting of toluene, sulfuric acid, and chloroform. In contrast, uncoated counterparts suffer immediate and irreversible damage. This work establishes a scalable approach to chemically resilient soft robots capable of reliable operation in corrosive liquid environments, opening new possibilities for long-term deployment in biomedicine, chemical tank inspection, polluted water remediation, and offshore infrastructure maintenance.

## Abstract 2

Enhanced chemical shielding capability of under liquid soft robots with superomniphobicity.

## Graphical abstract

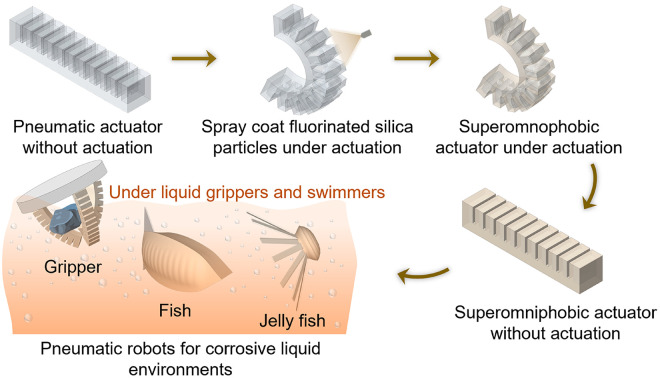

**New concepts**

We pioneer the application of superomniphobic coatings to significantly enhance the chemical shielding of soft robots in harsh corrosive liquid environments. This coating strategy effectively prevents wetting and provides robust protection against strong acids and organic solvents across diverse soft robotic platforms, including those fabricated from silicone elastomers, liquid crystal elastomers, and magnetic soft composites. Remarkably, the coating maintains excellent liquid repellency even under large deformations of up to 100% strain, ensuring reliable and repeatable performance. We further demonstrate the versatility of this approach through a range of soft robotic motions, including bending, stretching, crawling, and swimming, highlighting the potential of superomniphobic coatings to enable safe and durable operation of soft robots in extreme corrosive environments.

## 1. Introduction

Corrosive liquid environments, such as chemical tanks,^1^ polluted oceans,^2^ and hot springs,^3^ pose significant hazards but remain central to a wide range of industrial,^4^ environmental,^5^ and biomedical activities.^6^ Despite their potential, soft robotic systems^7^ designed to operate in such harsh conditions remain relatively underexplored. Compared to rigid robots, soft robots offer key advantages, including intrinsic compliance,^8–11^ adaptability to confined spaces,^12,13^ and safe interactions^14,15^ with delicate environments. In particular, soft swimming robots^14,16–19^ show great promise for applications such as hazardous chemical leak detection,^20^ inspection of pipelines^21^ and chemical storage tanks,^22^ and environmental monitoring^19^ in contaminated marine ecosystems and offshore oil platforms. Their ability to deform, navigate confined spaces, and traverse complex fluidic pathways, make them especially suited for these tasks. Recent advancements in stimuli-responsive soft robots,^23–26^ especially magnetically actuated systems,^27–29^ have further extended their utility to minimally invasive biomedical applications. These robots can navigate physiological environments, such as acidic gastric fluids or alkaline intestinal tracts, under wireless control with high precision and biocompatibility, minimizing the risk of tissue damage or contamination. Additionally, soft robots based on liquid crystal elastomers (LCEs) have demonstrated exceptional potential for high-temperature liquid environments,^9,10,24,30^ enabling untethered shape-morphing^31^ and functional deployment^32^ in thermally extreme or emergency scenarios, such as relief and rescue missions.

Soft robots are typically constructed from highly stretchable soft materials such as silicone rubber, LCEs,^33,34^ magnetic elastomer composites, dielectric elastomer,^35,36^ and hydrogels. These materials provide the compliance and deformability necessary for soft actuation. However, most of these systems are limited to operation in non-harsh environments, as many elastomers are susceptible to degradation under chemically harsh conditions. For example, pneumatic actuators may suffer from air leakage due to corrosion-induced damages, magnetic composites can warp, and LCEs tend to swell in the presence of organic solvents, resulting in structural deformation and compromised functionality.

Exposure to corrosive liquids, such as organic solvents (*e.g.*, toluene), strong acids (*e.g.*, sulfuric acid), or bases (*e.g.*, liquid sodium hydroxide), poses severe threats to soft robotic systems by accelerating chemical degradation, mechanical failure, and irreversible material damage. Acidic and alkaline media, in particular, can break down polymer chains, leading to cracking, swelling, and loss of mechanical integrity. These challenges significantly hinder the deployment of soft robots in critical applications involving chemical spills, industrial inspection, or hazardous environments. Therefore, imparting chemical shielding to soft robots is essential to prevent elastomer degradation and to ensure functionality and reliability in corrosive liquid environments.

One way to provide chemical shielding on the surface of the elastomer is making the elastomer surface superomniphobic (*i.e.*, repellent to virtually any liquid). Superomniphobic surfaces offer chemical shielding against a wide range of liquids – organic or aqueous, polar or nonpolar, Newtonian or non-Newtonian – by making droplets bead up, bounce and easily roll off from the surface. In order to fabricate superomniphobic surfaces, it is essential to have a good understanding of the fundamentals of wetting that lead to superomniphobicity. The primary measure of wetting of a liquid on a nontextured (*i.e.*, smooth) solid surface is the Young's contact angle *θ*.^37^ Typically, surfaces with low solid surface energy *γ*_sv_ result in high Young's contact angles.^38–40^ To design super-repellent surfaces, in addition to low solid surface energy, a surface texture (or roughness) is necessary.^41^ When a liquid droplet comes into contact with a textured solid surface, it displays an apparent contact angle θ*, which is different from the Young's contact angle *θ*. On the textured solid surface, the droplet can assume either the Wenzel state^42^ or the Cassie–Baxter state.^43^ In the Wenzel state, the liquid droplet completely penetrates the surface texture, while in the Cassie–Baxter state, there are pockets of air trapped within the surface texture beneath the droplet. The reduced solid–liquid contact area and the increased liquid–air contact area in the Cassie–Baxter state often allow droplets to display a very high apparent advancing and receding contact angles, 
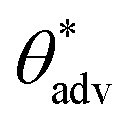
 and 
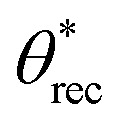
, as well as very low contact angle hysteresis 
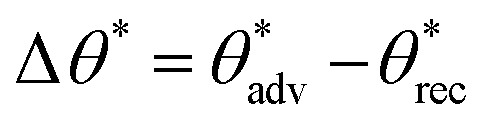
.^44^ Such low contact angle hysteresis results in high mobility of the droplets with very low sliding angles *ω* (the minimum angle by which the surface must be tilted relative to the horizontal for the droplet to slide).^45,46^ As a result, the Cassie–Baxter state is preferred for designing super-repellent surfaces with apparent contact angles θ* > 150° and sliding angles *ω* < 10°. Surfaces are considered superhydrophobic if they display θ* > 150° and *ω* < 10° for high surface tension liquids (*e.g.*, water),^47^ and superomniphobic if they display θ* > 150° and *ω* < 10° for high surface tension liquids as well as low surface tension liquids (*e.g.*, oils).^48^ Unlike superhydrophobic surfaces, re-entrant texture (*e.g.*, convex, overhang or undercut texture) is necessary to design superomniphobic surfaces.^38^ Many prior reports have demonstrated superomniphobicity through an appropriate combination of re-entrant texture and low solid surface energy. Superomniphobic surfaces display a plastron layer (*i.e.*, shiny air pockets when submerged in a liquid), indicative of a Cassie–Baxter state, with a wide range of liquids.^49,50^ Such Cassie–Baxter state against a broad spectrum of liquids, including organic, aqueous, polar, nonpolar, and even non-Newtonian liquids, enables effective protection and greatly expands the operational range of soft robots in harsh chemical environments.^51^

**Fig. 1:**
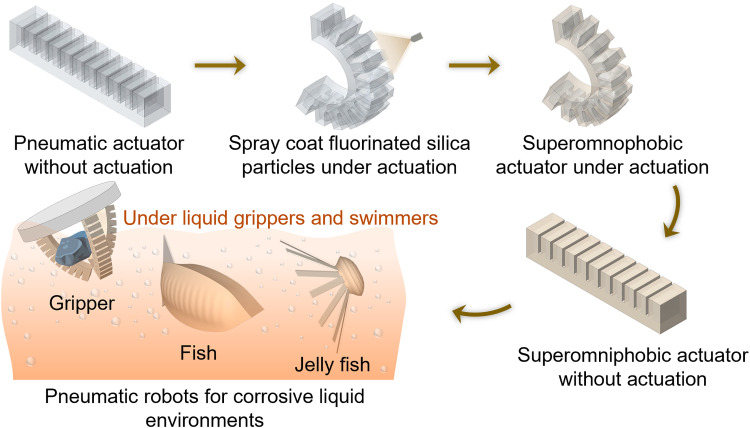
Enhanced chemical shielding with superomniphobicity. Schematic illustrating the fabrication of superomniphobic soft robots.

Building on this understanding, we develop soft robots with enhanced chemical shielding by imparting superomniphobicity to their surfaces. A simple, economical, and scalable approach for achieving superomniphobicity involves spray-coating fluorinated silica nanoparticles onto the elastomeric substrate.^52^ These particles create a re-entrant micro/nanotexture and impart low surface energy due to fluorination, together enabling robust superomniphobic behavior (Fig. 1). In our approach, pneumatic soft robots are first actuated into a deformed state, during which the coating is applied. This ensures uniform coverage and results in a superomniphobic layer that remains intact at both actuated and unactuated states. This robust coating enables various soft robotic platforms to operate safely and reliably in corrosive liquid environments by shielding the underlying elastomer from degradation. We expect that the superomniphobic coating enhances chemical resistance by maintaining an air layer at the solid–liquid interface and minimizing direct contact with aggressive fluids, regardless of their surface tension, viscosity, pH, or toxicity. We demonstrate that chemically shielded soft grippers can safely grasp and release solid objects in concentrated sulfuric acid, and bioinspired soft swimmers can navigate 70% nitric acid without damage. Furthermore, we demonstrate that the same strategy can be applied to other stimuli-responsive soft actuators, such as LCE ribbons and magnetically driven soft crawlers, for chemically shielded deformation and motion in organic solvents like toluene and chloroform. We envision that this strategy will advance the development of durable soft robots for long-term operation in harsh liquid environments across applications in food processing, healthcare, and the chemical industry.

## 2. Results and discussions

### 2.1 Characterization of superomniphobic coating

We fabricated bio-inspired soft robots, including gripper, fish, and jellyfish, using pneumatic soft actuators, and subsequently spray-coated them with low surface energy fluorinated silica nanoparticles to impart superomniphobicity (see SI, Section S1). To ensure uniform and robust surface coverage, the actuators were stretched during coating. This pre-strain allows the coating to remain intact and functional under repeated deformation and actuation (Fig. 1).

**Fig. 2:**
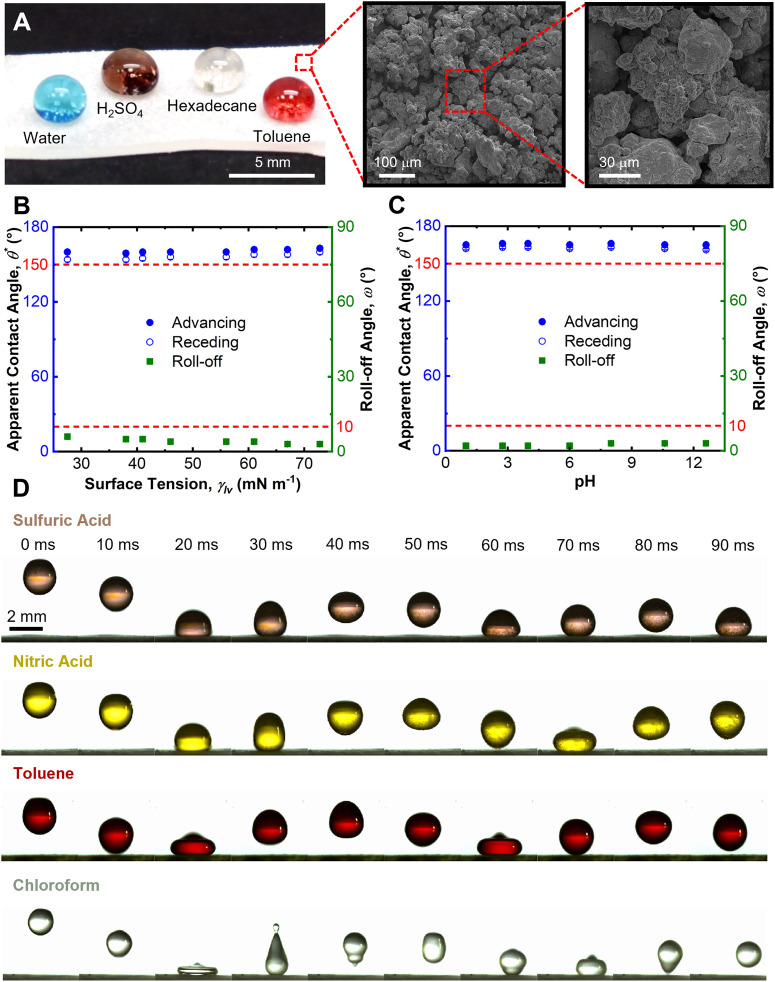
Characterization of superomniphobic coating. (A) Image (left) depicting the different liquid (*i.e.*, high surface tension liquid like water, corrosive liquid like sulfuric acid, low surface tension liquid like hexadecane, organic solvent like toluene) droplets beading up on a superomniphobic silicone sheet. SEM images depict the surface morphology of superomniphobic silicone sheet. (B) and (C) Droplet contact angles and roll off angles on superomniphobic silicone sheet as a function of liquid surface tension and pH, respectively. (D) Time-lapse images depicting droplets of sulfuric acid, nitric acid, toluene, and chloroform bouncing on a superomniphobic silicone sheet.

We investigated the surface morphology of the coated actuators using scanning electron microscopy (SEM; Fig. 2(A) and see Experimental section). SEM images revealed agglomerates of fluorinated silica particles forming a characteristic re-entrant texture. The combination of this texture and the low surface energy from fluorination rendered the surface superomniphobic. Wettability was characterized *via* contact angle goniometry using 20 µL droplets of various liquids with different surface tensions (Fig. 2(B)). The coated surfaces exhibited high advancing and receding contact angles (
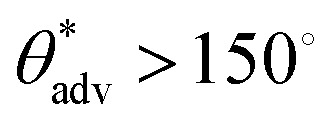
, 
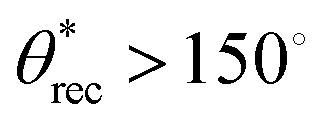
) and low roll-off angles (*ω* < 10°) for both high and low surface tension liquids, indicating the extreme repellency and low liquid adhesion (SI, Section S2 and Experimental Section) of our superomniphobic soft actuators to a wide range of liquids. For example, with water (*γ*_lv_ = 72.8 mN m^−1^), 
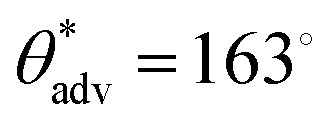
, 
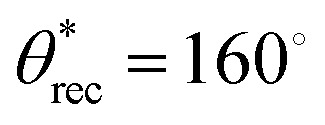
, and *ω* = 3°, while for concentrated nitric acid (*γ*_lv_ = 41.3 mN m^−1^), 
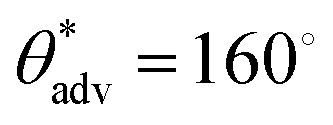
, 
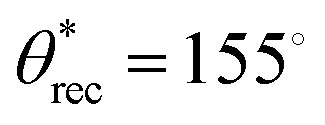
, and *ω* = 5°. Furthermore, the actuators also displayed extreme repellency to corrosive liquids of varying pH. For example, for pH = 1, 
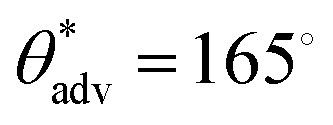
, 
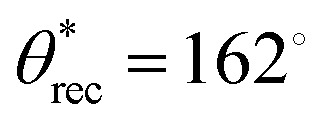
, *ω* = 1°; and for pH = 12.6, 
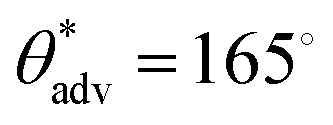
, 
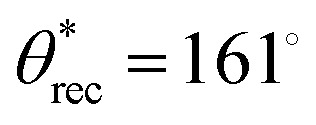
, *ω* = 3° (Fig. 2(C)). Finally, the superomniphobicity was visually confirmed by the ability of a wide range of liquid droplets, including concentrated sulfuric acid (*γ*_lv_ = 67.1 mN m^−1^), nitric acid (*γ*_lv_ = 41.3 mN m^−1^), and organic solvents like toluene (*γ*_lv_ = 28.4 mN m^−1^) and chloroform (*γ*_lv_ = 27.1 mN m^−1^), to easily bounce and roll off the surface without wetting (Fig. 2(D) and Movie S1, SI).

### 2.2 Superomniphobicity of soft actuators under strain

**Fig. 3:**
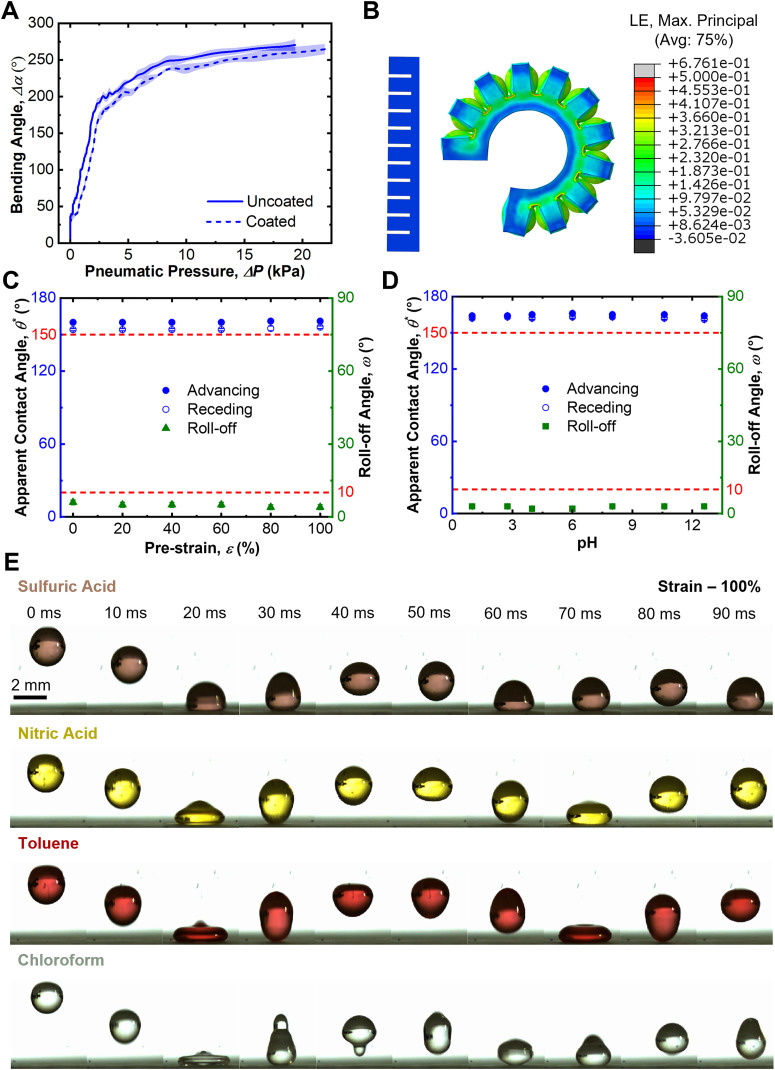
Superomniphobicity of soft actuator under strain. (A) Plot depicting bending angle of uncoated and superomniphobic soft actuator as a function of pneumatic pressure. (B) Image depicting strain distribution on soft actuator under actuation. (C) Droplet contact angles and roll off angles of hexadecane on a superomniphobic silicone sheet as a function of strain percentage. (D) Droplet contact angles and roll off angles of liquids with different pH on a superomniphobic silicone sheet at 100% strain. (E) Time-lapse images depicting droplets of sulfuric acid, nitric acid, toluene, and chloroform bouncing on a superomniphobic silicone sheet under 100% strain.

During operation, superomniphobic soft actuators must maintain both functional properties – mechanical actuation and extreme liquid repellency. To verify that the superomniphobic coating does not compromise actuation performance, we systematically evaluated the bending behavior of pneumatic soft actuators under increasing pneumatic pressure. As shown in Fig. 3(A), both coated and uncoated actuators exhibit similar trends: a rapid increase in bending angle at low pressure (*P* < 5 kPa), followed by saturation beyond 10 kPa. Finite element analysis (FEA) simulations (Fig. 3(B), see Experimental Section) closely match the experimental results, confirming that the superomniphobic coating does not affect actuation mechanics.

To ensure that superomniphobicity is preserved during deformation, we characterized wettability across a range of strains up to 100%, well beyond the maximum strain experienced during actuation, as determined by FEA. We measured advancing and receding contact angles and roll-off angles (using 20 µL droplets) of liquids with varying surface tension and pH (Fig. 3(C) and (D)). Across all strain levels, the actuators maintained high contact angles (>150°) and low roll-off angles (<10°). For example, with hexadecane (a representative low surface tension liquid; *γ*_lv_ = 27.5 mN m^−1^), 
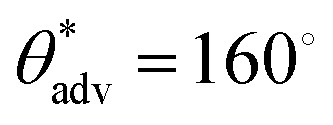
, 
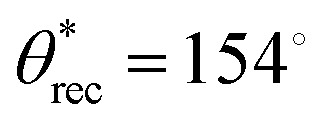
, *ω* = 6° at 0% strain, and 
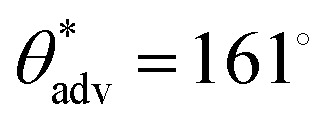
, 
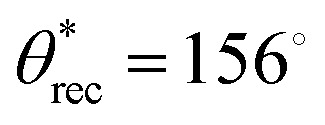
, *ω* = 4° at 100% strain. Similarly, extreme repellency was retained for corrosive liquids across pH values: 
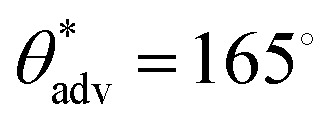
, 
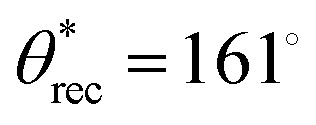
, *ω* = 3° for pH = 1, and 
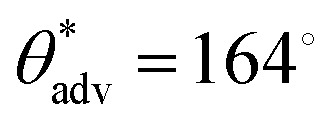
, 
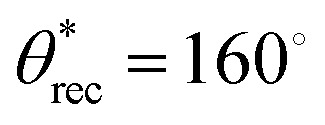
, *ω* = 3° for pH = 12.6 at 100% strain (Fig. 3(D)).

These results confirm the simultaneous robustness of both actuation and chemical repellency in the coated soft actuators. This is further visually confirmed by droplets of corrosive liquids (*e.g.*, sulfuric acid, nitric acid) and organic solvents (*e.g.*, toluene, chloroform) easily bouncing on and rolling off the actuators even at 100% strain (Fig. 3(E) and Movie S2, SI).

### 2.3 Performance of chemical shielding against harsh liquids

**Fig. 4:**
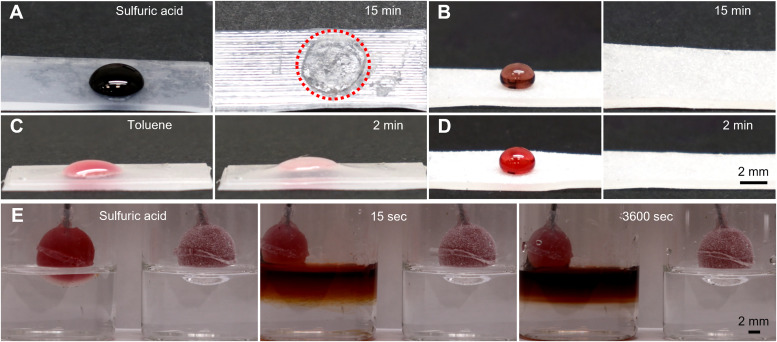
Chemical shielding performance of superomniphobic coated and uncoated elastomers. (A) & (B) Images depicting the surface damage on uncoated and superomniphobic silicone sheet, respectively after contacting 30 µl sulfuric acid droplets. (C) & (D) Images depicting the swelling on uncoated and superomniphobic silicone sheets, respectively after contacting 30 µl toluene droplets. (E) Time-lapse images depicting dyed water (red) leak from uncoated (left) silicone balloons due to damage propagation in the presence of concentrated sulfuric acid. Superomniphobic silicone balloon (right) displayed no water leak for at least 1 hour indicating the enhanced chemical shielding on the balloon offered by superomniphobic coating even under inflation.

The primary motivation for imparting superomniphobicity to soft actuators is to enhance their chemical shielding against harsh liquids. To demonstrate this effect, we exposed coated and uncoated actuators to concentrated sulfuric acid and toluene. As shown in Fig. 4(A), the surface of the uncoated actuator showed visible damage within 15 minutes of exposure to concentrated sulfuric acid, whereas the coated actuator exhibited no discernible change in appearance or color (Fig. 4(B)). Similarly, exposure to toluene caused significant swelling of the uncoated actuator within 2 minutes (Fig. 4(C)), while the superomniphobic-coated actuator remained unaffected (Fig. 4(D)).

To further highlight the protective function of the coating, we fabricated balloon-like test samples by molding the same elastomer used for the soft actuators, then spray-coated them to render the surface superomniphobic. Balloons filled with dyed water were immersed in concentrated sulfuric acid. The uncoated balloon failed within 15 seconds, resulting in immediate leakage of the dyed water. In contrast, the coated balloon remained intact, with no sign of leakage or surface degradation (Fig. 4(E) and Movie S3, SI).

These results confirm that the superomniphobic coating provides effective and robust chemical shielding for soft robotic systems in aggressive liquid environments.

### 2.4 Underwater object manipulation and swimming in harsh environments

Next, leveraging the advantages of superomniphobic-coated soft actuators, including chemical shielding, safe interaction, and fast response, we demonstrate their utility in two representative applications: (1) noninvasive, delicate manipulation using a three-finger soft gripper, and (2) bioinspired swimming robots, including fish- and jellyfish-inspired designs, capable of reliable operation in corrosive liquid environments. To assess corrosion resistance, we conducted functional tests in highly acidic conditions, demonstrating that chemically shielded soft robots retain full functionality without visible degradation during direct and prolonged exposure.

**Fig. 5:**
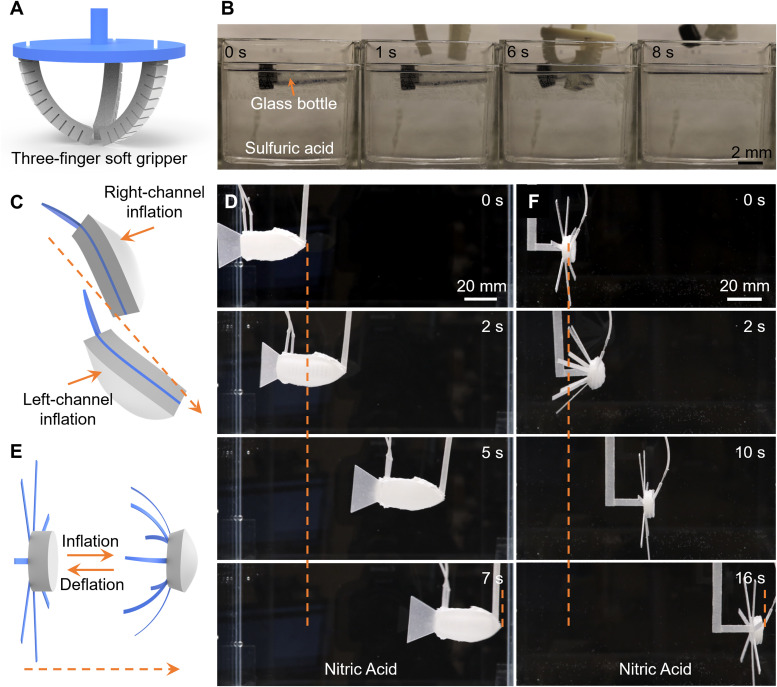
Demonstrations of pneumatic-driven gripper and swimming robots. (A) Schematic design of a three-finger pneumatic-driven soft gripper. (B) Time-lapse images showing the grasping process of the gripper in sulfuric acid. Scale bar: 2 mm. (C) Schematic design of a bidirectional bending, fish-inspired pneumatic soft swimming robot. (D) Time-lapse images showing the undulatory swimming process of the fish-inspired soft robot in nitric acid. Scale bar: 20 mm. (E) Schematic design of a jellyfish-inspired pneumatic soft swimming robot. (F) Time-lapse images showing the swimming process of the jellyfish-inspired soft robot in nitric acid. Scale bar: 20 mm.

Fig. 5(A) shows the schematic of a three-finger superomniphobic soft gripper, composed of pneumatic bending actuators mounted on a 3D-printed holder for stable grasping in corrosive liquid environments. Upon inflation, the actuators bend uniformly toward the central axis. Time-lapse images (Fig. 5(B)) show the gripper gently capturing and lifting a floating glass bottle from 40% sulfuric acid within 8 seconds (Fig. S3 and Movie S4, SI). Fig. 5(C) presents the schematic of a fish-inspired soft swimming robot with a bidirectional bending design. It consists of a fish-shaped polyester sheet sandwiched between two pneumatic bending actuators. A floating pad connected *via* a thin rod maintains the swimming height and balance. By alternating actuation, the robot propels forward in concentrated nitric acid, mimicking undulatory motion (Fig. 5(D) and Movie S5, SI). The average relative swimming speed is ∼0.3 body length per second (BL per s), exceeding previously reported fish-like soft swimmers.^53,54^ Inspired by the rhythmic contraction and relaxation motions of jellyfish, we also developed a jellyfish-inspired robot composing a 3D doming actuator and seven attached plastic paddles to enhance thrust. Upon inflation, the actuator contracts, followed by passive relaxation upon deflation (Fig. 5(E)). Time-lapse images (Fig. 5(F)) and video (Movie S6, SI) show robust forward swimming in nitric acid, achieving a high relative swimming speed is ∼1.5 BL s^−1^, surpassing most jellyfish-inspired soft robots reported to date.^15,55,56^

These demonstrations highlight the effectiveness of superomniphobic coatings in enabling chemically shielded soft robots to perform complex tasks safely and reliably in extreme environments, with potential applications in chemical processing, polluted marine ecosystems, and environmental monitoring.

### 2.5 Chemically shielded stimuli-responsive soft actuators

**Fig. 6:**
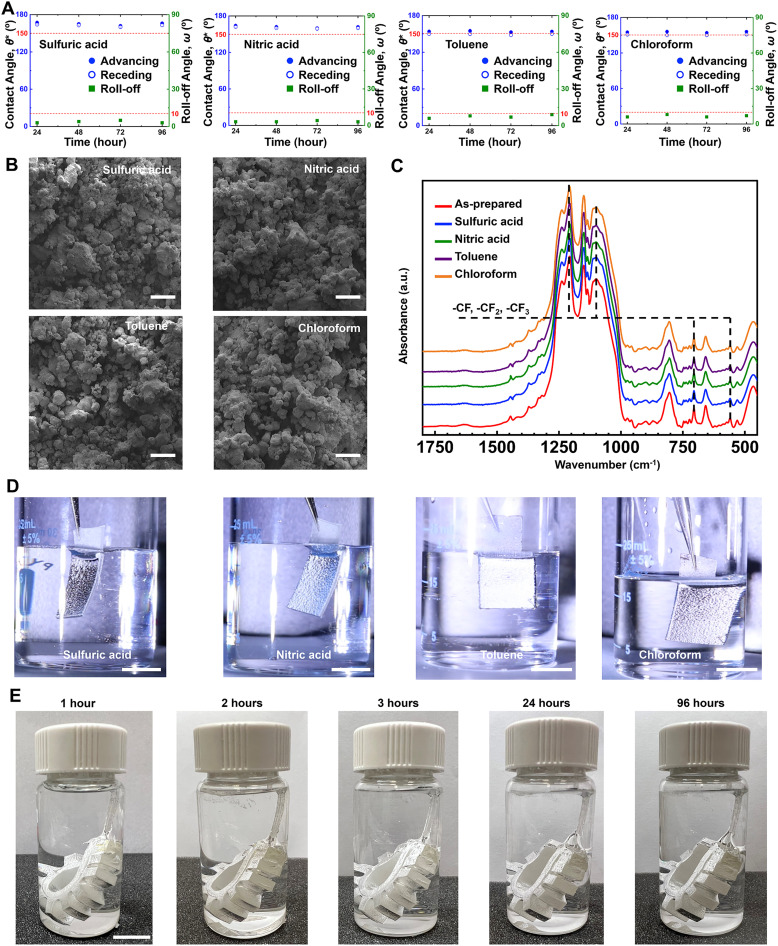
Effectiveness of the superomniphobic coatings on soft actuators. (A) Apparent contact angles and roll-off angles of hexadecane, (B) SEM images depicting surface morphology, (C) FTIR spectra depicting surface chemistry, and (D) Images depicting plastron layer, for pneumatic actuator after immersion in sulfuric acid, nitric acid, toluene, and chloroform. In (A), top dotted line represents 150° contact angle, and bottom dotted line represents 10° roll-off angle. (E) Snapshots of the superomniphobic pneumatic actuator immersed in sulfuric acid. In (B), (D) and (E), scale bars represent 100 µm, 1 cm and 1 cm, respectively.

To assess our superomniphobic coatings on actuators under harsh environments, we subjected the pneumatic, LCE, and magnetic actuators to a series of chemical, thermal, and actuation cycle tests. All actuators maintained their superomniphobicity upon cyclic heating and cooling up to 200 °C (SI, Section S4), as well as up to 500+ actuation cycles (SI, Section S5). Furthermore, we evaluated the chemical robustness of the coating through a series of tests with harsh liquids. For example, there was no significant change in apparent contact angles and roll-off angles of hexadecane even after sliding 1000 droplets of sulfuric acid, nitric acid, toluene or chloroform on the actuator surface (SI Section 6). Even upon immersing the actuator in sulfuric acid, nitric acid, toluene, and chloroform, it retained superomniphobicity for 96 h, as evident from no significant change in apparent contact angles and roll-off angles of hexadecane (Fig. 6(A)). The retention of superomniphobicity is also evident from no significant change in surface morphology (Fig. 6(B)) compared to the as-prepared superomniphobic actuator surfaces (Fig. 2(A)). Furthermore, FTIR spectra confirmed no significant change in surface chemistry before and after immersion of the surfaces in different harsh liquids (Fig. 6(C)). Even after prolonged immersion, fluorocarbon functional groups are retained, as evident from the strong absorption peaks around 1200 cm^−1^ corresponding to the –CF_2_ and –CF_3_ group stretching, as well as absorption peaks around 575 cm^−1^, 730 cm^−1^ and 1120 cm^−1^ corresponding to the vibration of the C–F bond of –CF_2_ and –CF_3_ groups.^57–59^ As a result of the intact surface morphology and surface chemistry of the superomniphobic coating, the immersed surfaces sustained a stable plastron layer (shiny surface indicative of trapped air pockets) in different harsh liquids that have enthalpically favorable interactions with the underlying substrate (Fig. 6(D)). Snapshots of the superomniphobic pneumatic actuator immersed in sulfuric acid at different times also confirm the retention of physical integrity and the plastron layer (Fig. 6(E)). Collectively, these results highlight the effectiveness of the superomniphobic coatings on soft actuators.

**Fig. 7:**
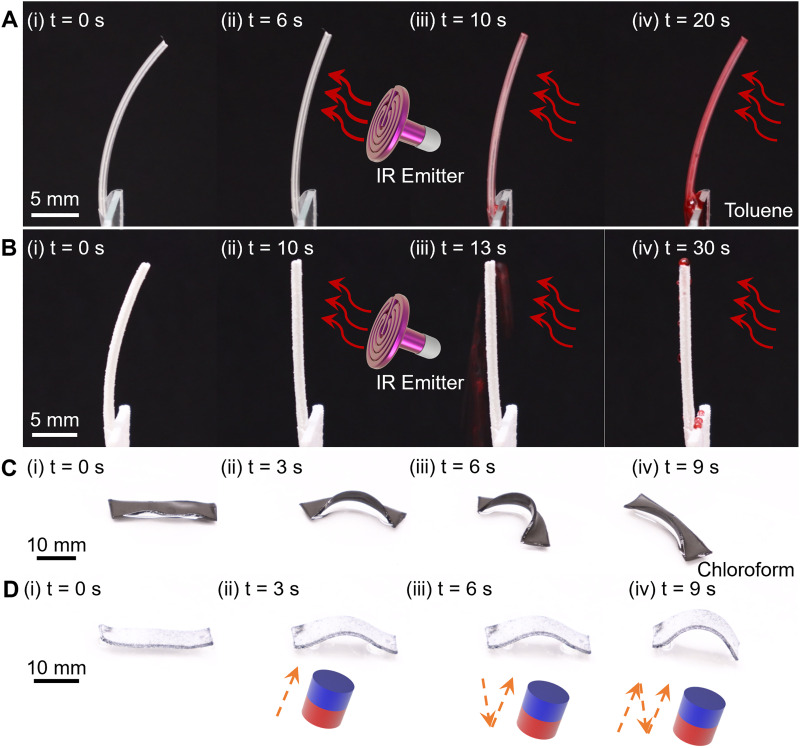
Demonstrations of LCE-based actuators and magnetic-driven crawlers. (A) and (B) Time-lapse images showing the bending process of LCE ribbons exposed to continuous toluene droplets under heating using a remote IR emitter. (A) Uncoated ribbon; (B) coated ribbon. (C) and (D) Time-lapse images showing the crawling performance of magnetic-driven soft actuators in a chloroform environment under magnetic actuation. (C) Uncoated actuator; (D) coated actuator.

To further demonstrate the versatility and chemical robustness of the superomniphobic coating, we explored various robotic applications in harsh liquid environments (*e.g.*, toluene and chloroform) using two distinct stimuli-responsive soft actuators, LCE-based and magnetically responsive soft composites (Fig. 7). Fig. 7(A) and (B) show the bending behavior of uncoated and coated LCE ribbons under infrared (IR) heating while being continuously exposed to toluene droplets (Movie S7, SI). Initially, both ribbons are in a curved configuration; upon heating with a remote IR emitter at 60 °C, they straighten within 10 s (Fig. 7(A)-(ii) and (B)-(ii)). However, under continuous exposure to red-dyed toluene droplets, the uncoated LCE ribbon becomes fully saturated, rapidly losing its two-way shape-memory behavior and actuation capability. In contrast, the coated ribbon retains its straightened form as all toluene droplets roll off without wetting or absorption (Fig. 7(A)-(iii), (iv) and (B)-(iii), (iv)).

Fig. 7(C) and (D) show the time-lapse crawling of uncoated and coated magnetically actuated soft robots in shallow chloroform (Fig. S7 and Movie S8, SI). At *t* = 0 s, the uncoated crawler shows initial edge warping, whereas the coated crawler remains flat. Upon magnetic actuation *via* a robotic arm-mounted permanent magnet, the coated robot undergoes repeatable bending–unbending motions to crawl forward without visible degradation (Fig. 7(D)-(ii)–(iv)). In contrast, the uncoated crawler suffers rapid chemical degradation, warping significantly and ultimately losing magnetic responsiveness within 9 s (Fig. 7(C)-(ii)–(iv)).

By preventing corrosive absorption and surface degradation, the coating preserves functionality and enables sustained actuation in otherwise prohibitive environments. This enhanced solvent resistance broadens the applicability of soft robots for use in chemically aggressive settings, including chemical manufacturing, pharmaceutical processing, and solvent-rich biomedical environments.

## 3. Conclusions

We demonstrated a range of soft robots with enhanced chemical shielding, capable of reliable and robust operation in harsh liquid environments. By integrating spray-coated superomniphobic surfaces onto both pneumatic-driven and stimuli-responsive soft actuators, we achieved exceptional liquid repellency across a broad spectrum of corrosive and organic solvents, including concentrated sulfuric and nitric acids, toluene, and chloroform. The superomniphobic coating preserved both mechanical functionality and surface repellency under significant deformation and strain. This chemical shielding enabled soft grippers and bioinspired swimming robots to perform challenging tasks, such as noninvasive object manipulation and undulatory swimming, in corrosive media without degradation or performance loss. We further extended this approach to LCE-based and magnetically actuated soft robots, demonstrating temperature- and magnetic-driven motion in solvent-rich environments. This work establishes a simple, scalable, and versatile strategy to protect soft robotic systems, paving the way for long-term deployment in challenging liquid environments relevant to chemical processing, biomedicine, environmental monitoring, and marine exploration. The integration of robust chemical shielding with soft robotics marks a key step toward real-world applications requiring both material resilience and environmental adaptability.

Despite these promising advances, several limitations remain. First, while the chemically shielded soft robots exhibited reliable performance and large deformations in simple, unimodal motion, more complex shape transformations and multimodal locomotion remain to be achieved for navigating complicated harsh liquid environments. These could be enabled through the integration of advanced actuation strategies and control architectures. Second, the durability of the superomniphobic coating is currently limited: while the chemical durability is impressive, abrasion can damage the coating. Future work may extend the durability under abrasion through optimization of the coating formulation, surface adhesion, and spray-coating process.

## 4. Experimental section

### 4.1 Fabrication of pneumatic bending actuators

All pneumatic bilayer actuators were fabricated using a standard molding and demolding process commonly employed for fluid-driven soft actuators. Ecoflex 00–50 (Smooth-On Inc.) was used as the elastomeric material for the homogeneous bilayer structures. The bilayer, consisting of a flat thin layer and a pneumatic channel layer, was formed by casting the elastomer into 3D-printed molds (Stratasys Objet260) and curing in an oven at 50 °C for 12 hours. After curing, the layers were demolded and bonded together using Smooth-On SIL-Poxy silicone adhesive, followed by an additional curing step at 50 °C for 3 hours to ensure strong adhesion.

### 4.2 Fabrication of superomniphobic actuators

To render the surface of the soft actuator superomniphobic, we employed a spray-coating method using fluorinated fumed silica particles. These particles were synthesized *via* liquid-phase silanization by dispersing 500 mg of 7 nm fumed silica (Sigma-Aldrich) in 20 mL of hexane containing 0.5 mL of heptadecafluoro-1,1,2,2-tetrahydrodecyl trichlorosilane (Gelest Inc.). The reaction mixture was stirred for three days to ensure complete surface functionalization. The resulting fluorinated silica particles were then spray-coated onto the actuator surface to impart a thin layer of superomniphobic coating (see SI, Section S1).

### 4.3 FEA simulation

To investigate the bending deformation of pneumatic soft actuators, 3D models were created using SolidWorks and analyzed with the commercial finite element analysis software Abaqus/CAE, employing the Abaqus/Standard solver. The actuator geometries were imported into Abaqus/CAE as STL files and meshed using solid quadratic tetrahedral elements (C3D10H). A mesh refinement study was conducted to ensure the accuracy of the simulation results. The elastomer material (Ecoflex 00-50) was modeled as a hyperelastic, isotropic material using the Yeoh model, with the strain energy density function defined as:1
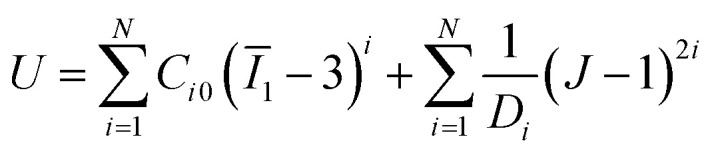
where *Ī*_1_ = tr[dev(***FF***)^T^], *J* = det(***F***), and *F* is the deformation gradient. The parameters *C*_*i*0_ and *D*_*i*_ are material constants. In our model, *N* = 3, *C*_10_ = 0.019, *C*_20_ = 0.0009, *C*_30_ = −4.75 × 10^−6^, and *D*_1_ = *D*_2_ = *D*_3_ = 0.^60^ Static simulations were performed by applying internal pressure loads to the cavity surfaces of actuators.

### 4.4 Contact angle and roll-off angle measurements

The contact angles and roll-off angles of liquid droplets on the fabricated surfaces were measured using a contact angle goniometer (Ramé-Hart 260). The advancing contact angle and receding contact angle were measured by increasing and decreasing, respectively, the volume of a droplet on the surface using a micrometer syringe (Gilmont). The roll-off angles were measured by slowly tilting the surface until the droplet (∼10 µL) started sliding on the surface. At least six measurements were performed on each surface at spatially distinct locations.

### 4.5 Normal and lateral adhesion measurements

Normal and lateral adhesion forces on the actuator surfaces were quantified using a force tensiometer (Krüss K100) with 10 µL water droplets. A droplet was mounted on the probe and brought into contact with each surface before initiating either normal or lateral displacement at a constant velocity of 5 µm s^−1^. During these controlled displacements, the instrument recorded the corresponding adhesion forces as the droplet detached or slid. For each sample, at least five measurements were performed at spatially distinct positions, and the averaged values are reported as the representative normal and lateral adhesion forces.

### 4.6 Morphology characterization

Surface morphology was characterized by using a scanning electron microscope (SEM; Thermofisher Phenom Pharos) at 5 kV. The samples were sputter coated with a thin film of gold prior to imaging (Cressington 108). Coating thickness was determined using a confocal laser scanning microscope (Keyence VKx1100).

### 4.7 Bouncing droplets

Movies of bouncing droplets were obtained using a high-speed camera (Fastcam Mini AX200) at 500 frames per second.

### 4.8 Fabrication of soft swimmers

The fish tail for the fish swimming robot and the swimming paddles for the jellyfish swimming robot were fabricated from thin polyester sheets, which were cut using a laser cutter. These components were embedded into the middle layer of the bidirectional bending actuators along the body axis in the fish design and around the edge of the circular structure in the jellyfish design. We fabricated and tested at least five independent samples.

### 4.9 Fabrication of LCE soft actuators

The LCE soft actuators were fabricated using a two-stage reaction process as described in previous work. Specifically, 2.0 g of 1,4-bis-[4-(3-acryloyloxypropyloxy)benzoyloxy]-2-methylbenzene (RM257; Wilshire Company, 95%) was fully dissolved in 0.7 g of toluene (Sigma-Aldrich, 99.8%) at 85 °C under continuous stirring. Subsequently, 0.012 g of (2-hydroxyethoxy)-2-methylpropiophenone (HHMP; Sigma-Aldrich, 98%), 0.42 g of 2,2′-(ethylenedioxy)diethanethiol (EDDET; Sigma-Aldrich, 95%), and 0.19 g of pentaerythritol tetrakis(3-mercaptopropionate) (PETMP; Sigma-Aldrich, 95%) were added to the solution. The mixture was stirred at 85 °C for 20 minutes. After cooling to room temperature, 0.006 g of dipropylamine (DPA; Sigma-Aldrich, 98%) was added as a catalyst, and the solution was stirred for an additional 3 minutes. The resulting prepolymer solution was poured into a mold and allowed to react overnight at room temperature. The sample was then left in the mold and dried at 75 °C for 24 hours. The resulting LCE ribbon was mechanically stretched to approximately 150% of its original length and subsequently exposed to UV irradiation for 10 minutes to complete the crosslinking process. We fabricated and tested at least five independent samples.

### 4.10 Fabrication of magnetic-driven soft crawlers

The soft magnetic composite was fabricated by blending uncured Ecoflex 00-50 silicone elastomer (Smooth-On) with neodymium–iron–boron (NdFeB) magnetic microparticles (MQFP-B + 20174-088, Magnequench). Parts A and B of Ecoflex 00-50 were mixed in a 1 : 1 weight ratio, followed by the addition of NdFeB particles at a concentration of 70 wt%. The mixture was thoroughly stirred and degassed under vacuum for 5 minutes to remove entrapped air bubbles. The resulting composite was cast into a rectangular mold (length: 20 mm; width: 10 mm; height: 1 mm) fabricated using Stratasys Objet260. After curing, the rectangular samples were axially magnetized by placing them between the poles of a GMW 3472-70 electromagnet and applying a uniform magnetic field of 1.75 T for 3 minutes, aligning and magnetizing the embedded particles. We fabricated and tested at least five independent samples.

## Author contributions

H. Q., S. V., Y. C., J. Y., and A. K. K. developed the concept and designed the experiments. H. Q., S. V., and Y. C. fabricated and characterized the prototypes. H. Q., S. V., Y. C., M. J. Z., and P.S. conducted experiments. Y. C. conducted finite element analysis simulations. H. Q. and M. J. Z. drafted the manuscript. J. Y. and A. K. K. revised the manuscript. All the authors contributed to the discussion, data analysis, and editing of the final manuscript.

## Conflicts of interest

The authors declare no conflict of interest.

## Supplementary Material

MH-013-D5MH01593F-s001

MH-013-D5MH01593F-s002

MH-013-D5MH01593F-s003

MH-013-D5MH01593F-s004

MH-013-D5MH01593F-s005

MH-013-D5MH01593F-s006

MH-013-D5MH01593F-s007

MH-013-D5MH01593F-s008

MH-013-D5MH01593F-s009

## Data Availability

The data supporting this article have been included as part of the supplementary information (SI). Supplementary information is available. See DOI: https://doi.org/10.1039/d5mh01593f.
