# Supplementary material for: Enhancing soft robots with chemical shielding for harsh corrosive liquid environments
Source: Mater Horiz. 2025 Dec 10;13(5):2334–46. doi: 10.1039/d5mh01593f (PMC12709453; doi:10.1039/d5mh01593f)
Supplement: MH-013-D5MH01593F-s001 [file MH-013-D5MH01593F-s001.pdf]

Supplementary Information (SI) for Materials Horizons.
This journal is © The Royal Society of Chemistry 2025

Supplementary Information

Enhancing Soft Robots with Chemical Shielding for Harsh Corrosive Liquid Environments

Haitao Qing[1,†], Sravanthi Vallabhuneni[1,†], Yinding Chi[1,†], Mohammad Javad Zarei[1,†], Pouya Sharbati[1], Haoze Sun[1], Jie Yin[1,*], and Arun Kumar Kota[1,*]

H. Qing, S. Vallabhuneni, Y. Chi, M. J. Zarei, P. Sharbati, H. Sun, J. Yin and A. K. Kota

[1]Department of Mechanical and Aerospace Engineering, North Carolina State University, Raleigh, NC 27695, USA.

[†]These authors contributed equally to this work.

[*]Corresponding Author. E-mail: jyin8@ncsu.edu and akota2@ncsu.edu

This supplementary information includes:

Figure S1-S7

Movie legends for Movie S1 to S8

Section 1: Influence of silanization parameters on superomniphobicity

We investigated the effect of silanization temperature (18–50°C) and silane concentration (2.5–25 vol% silane in solvent) on the fluorination of silica nanoparticles used in the superomniphobic coating. Contact-angle measurements on coated substrates indicated that both temperature and silane concentration had no significant influence on wetting properties in the range investigated. Static, advancing, receding, and roll-off angles were consistent across the 18–50°C range (Figure S1A), indicating that room-temperature silanization is sufficient to achieve superomniphobicity. Similarly, static, advancing, receding, and roll-off angles were consistent across the 2.5–25 vol% range (Figure S1B), indicating that silanization with 2.5 vol% concentration is sufficient to achieve superomniphobicity.

We used the minimal coating thickness sufficient to provide robust chemical shielding without impairing actuator flexibility or responsiveness. To determine the coating thickness, we conducted profilometry of a masked step edge (Figure S1C). The resulting profile confirmed a uniform, thin fluorinated layer with a thickness of ~245 µm.

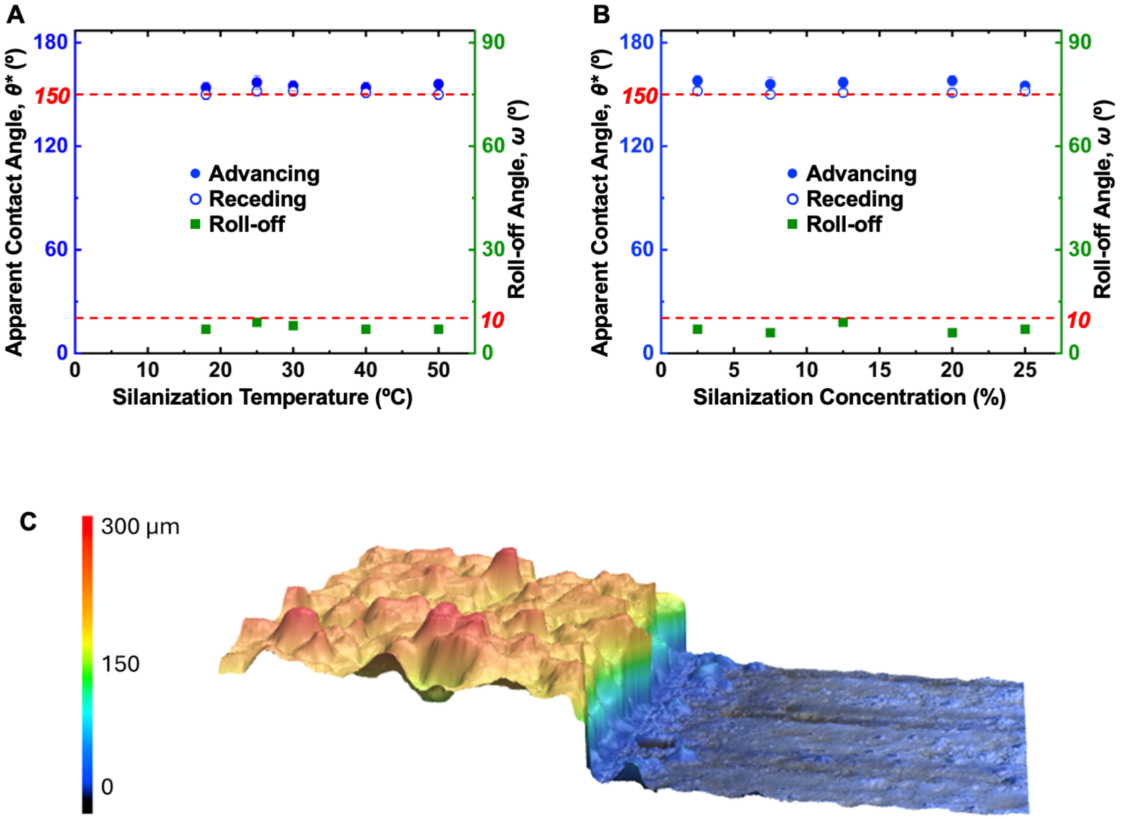

Figure S1. Optimization of silanization parameters for achieving robust superomniphobicity. **(A)** Apparent contact angles and roll-off angles of hexadecane for coatings prepared at different silanization temperatures. **(B)** Apparent contact angles and roll-off angles of hexadecane for coatings prepared at different silane concentrations. In A and B, top dotted line represents 150° contact angle, and bottom dotted line represents 10° roll-off angle. **(C)** 3D profile of a masked step edge confirming a uniform, thin fluorinated layer with a thickness of 245±20 μm.

Section 2: Normal and lateral adhesion force measurement on actuator surfaces

We evaluated the normal adhesion force (i.e., maximum force when a droplet detaches from the solid in the perpendicular direction) and lateral adhesion force (i.e., force required for a droplet to slide parallel to the solid) on uncoated and coated actuator surfaces using a Krűss K100 force tensiometer with 10 μL water droplets. All the coated superomniphobic actuator surfaces displayed very low normal adhesion forces, while the uncoated surfaces consistently displayed substantially higher normal adhesion forces (Figure S2A). Similarly, the coated surfaces displayed very low lateral adhesion forces, while the uncoated surfaces displayed substantially higher lateral adhesion forces (Figure S2B-D). At least five measurements were performed at spatially distinct positions on each sample.

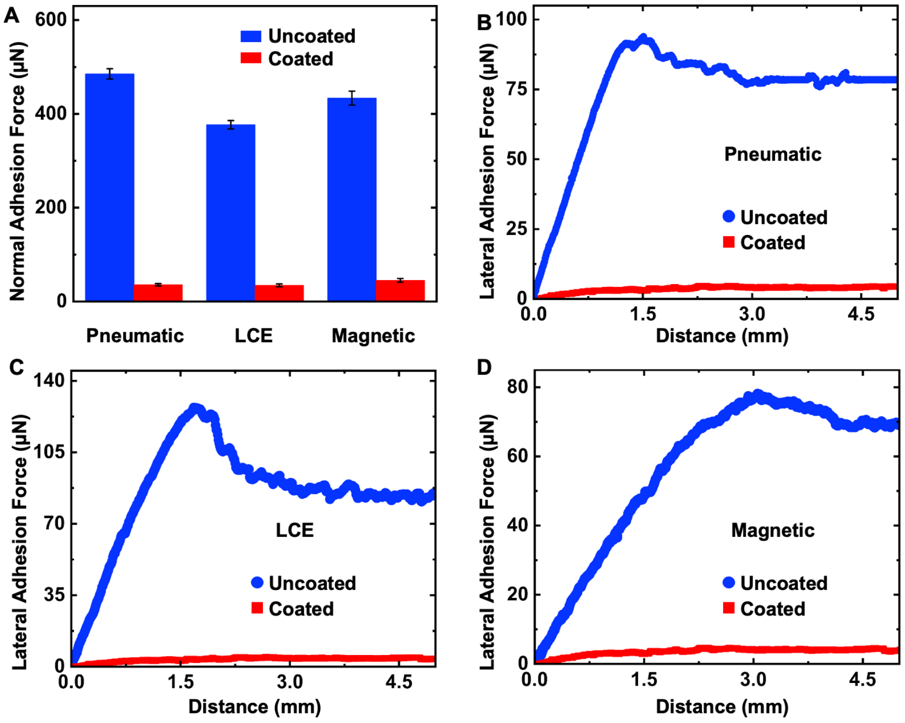

Figure S2. Normal and lateral adhesion forces on uncoated and coated actuator surfaces. (A) Normal adhesion forces measured using 10 μL water droplets on uncoated and coated pneumatic, LCE and magnetic surfaces. Lateral adhesion forces on coated and uncoated **(B)** pneumatic, **(C)** LCE, and **(D)** magnetic actuator surfaces.

Section 3: Grasping force test of the coated and uncoated pneumatic soft gripper

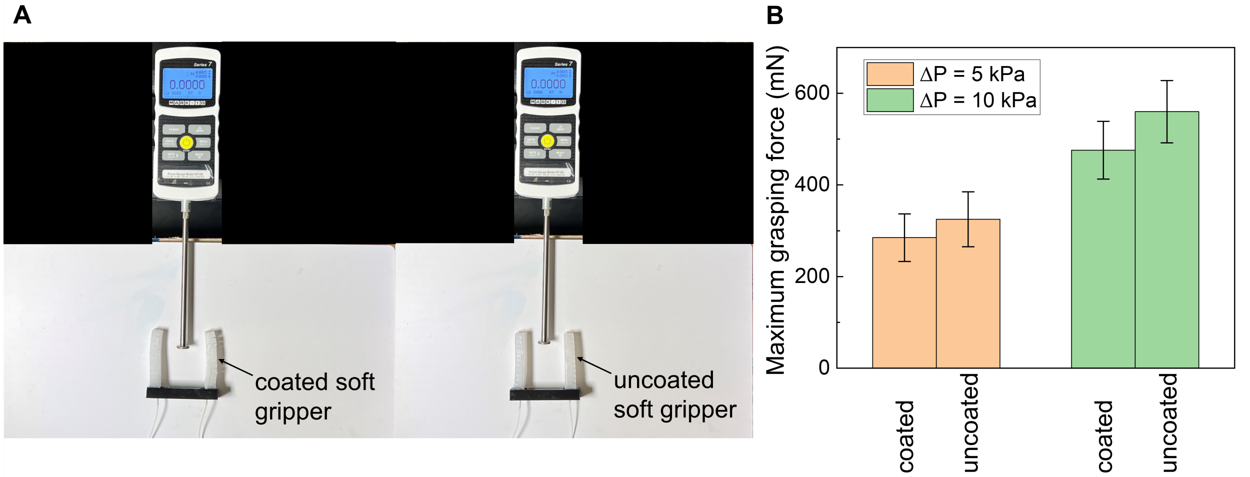

Figure S3. Grasping force measurement of soft grippers before and after coating. (A) Setup of grasping force measurement when ΔP = 0. **(B)** Pull-out force comparison of soft grippers before and after coating with different pneumatic pressures.

Section 4: Thermal stability of superomniphobic actuators

To evaluate thermal stability, we subjected the coated actuators to elevated temperatures up to 200°C for 1 h, and then cooled them down to ambient temperature. Then, we measured the advancing and receding contact angles, as well as the roll-off angles, using 10 μL droplets of hexadecane (Figures S3A-S3C). Our results indicate that all actuators maintained their superomniphobicity up to 200°C. Furthermore, SEM images of the heated and cooled superomniphobic actuator surfaces (Figure S3D-S3F) revealed no morphological changes compared to the as-prepared superomniphobic actuator surfaces (Figure 2A), confirming that the coatings are intact.

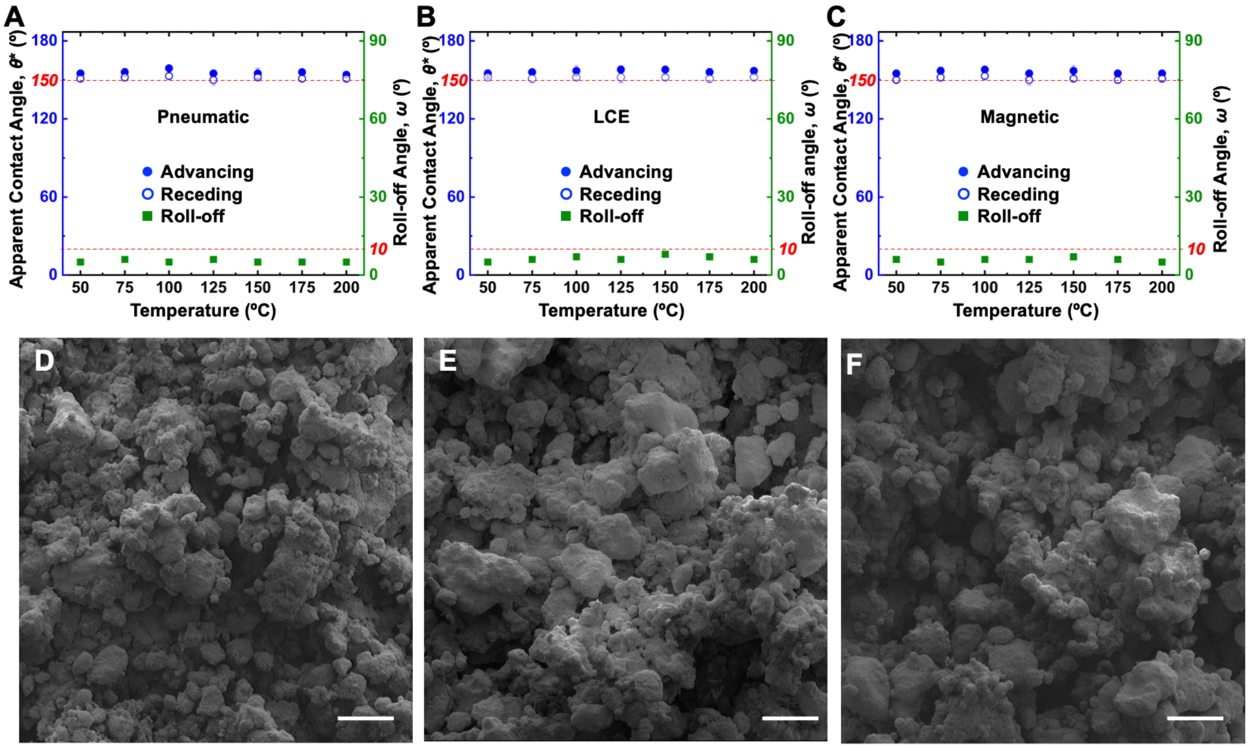

Figure S4. Thermal stability of the superomniphobic actuators. Apparent contact angles and roll-off angles of hexadecane on **(A)** pneumatic, **(B)** LCE, and **(C)** magnetic actuators after heating to 200 °C for 1 hour, and cooling to ambient temperature (top dotted line: 150° contact angle; bottom dotted line: 10° roll-off angle). SEM images of the **(D)** pneumatic, **(E)** LCE, and **(F)** magnetic actuator surfaces after heating to 200 °C for 1 hour, and cooling to ambient temperature. Scale bar represents 100 μm.

Section 5: Superomniphobicity after multiple actuation cycles

We subjected all actuators – pneumatic, LCE, and magnetic – to 500 consecutive actuation cycles, and measured the advancing and receding contact angles as well as the roll-off angles, using 10 μL droplets of hexadecane (Figure S4A-S4C). Our results indicate that all actuators maintained their superomniphobicity for at least 500 actuation cycles. Furthermore, SEM images of the superomniphobic actuator surfaces after 500 actuation cycles (Figure S4D-S4F) revealed no morphological changes compared to the as-prepared superomniphobic actuator surfaces (Figure 2A), confirming that the coatings are intact.

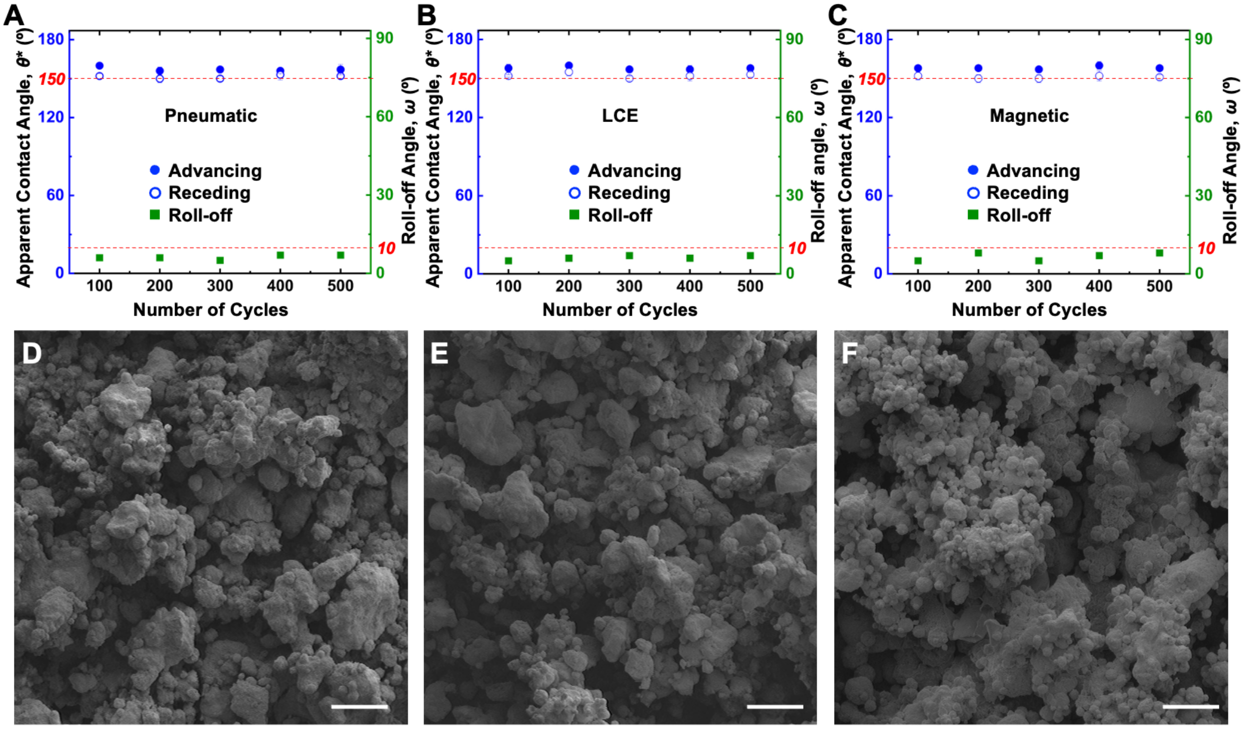

Figure S5. Superomniphobicity after multiple actuation cycles. Apparent contact angles and roll-off angles of hexadecane on **(A)** pneumatic, **(B)** LCE, and **(C)** magnetic actuators after 500 actuation cycles (top dotted line: 150° contact angle; bottom dotted line: 10° roll-off angle). SEM images of the **(D)** pneumatic, **(E)** LCE, and **(F)** magnetic actuator surfaces after 500 actuation cycles. Scale bar represents 100 μm.

Section 6. Superomniphobicity after repeated droplet sliding

We subjected the actuators surfaces to repeated corrosive droplet sliding, and measured the advancing and receding contact angles as well as the roll-off angles, using 10 μL droplets of hexadecane. For example, after sliding 1000 droplets of sulfuric acid, nitric acid, chloroform or toluene across the superomniphobic surface of the pneumatic actuator, the advancing, receding and roll-off hexadecane contact angles remained unchanged (Figures S5A-S5D), demonstrating that the coating preserves its superomniphobicity even after repeated corrosive droplet sliding.

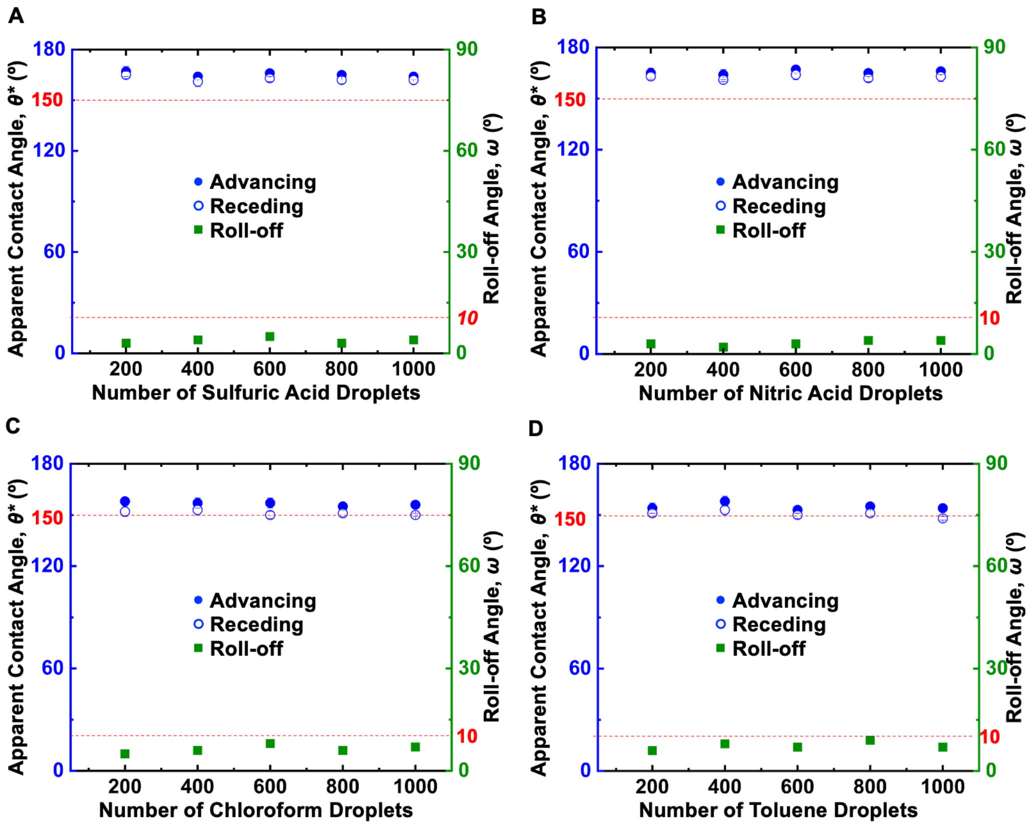

Figure S6. Superomniphobicity after repeated droplet sliding. Apparent contact angles and roll-off angles of hexadecane after sliding ~1000 droplets of **(A)** sulfuric acid, **(B)** nitric acid**, (C)** chloroform, and **(D)** toluene across the superomniphobic pneumatic actuator surface.

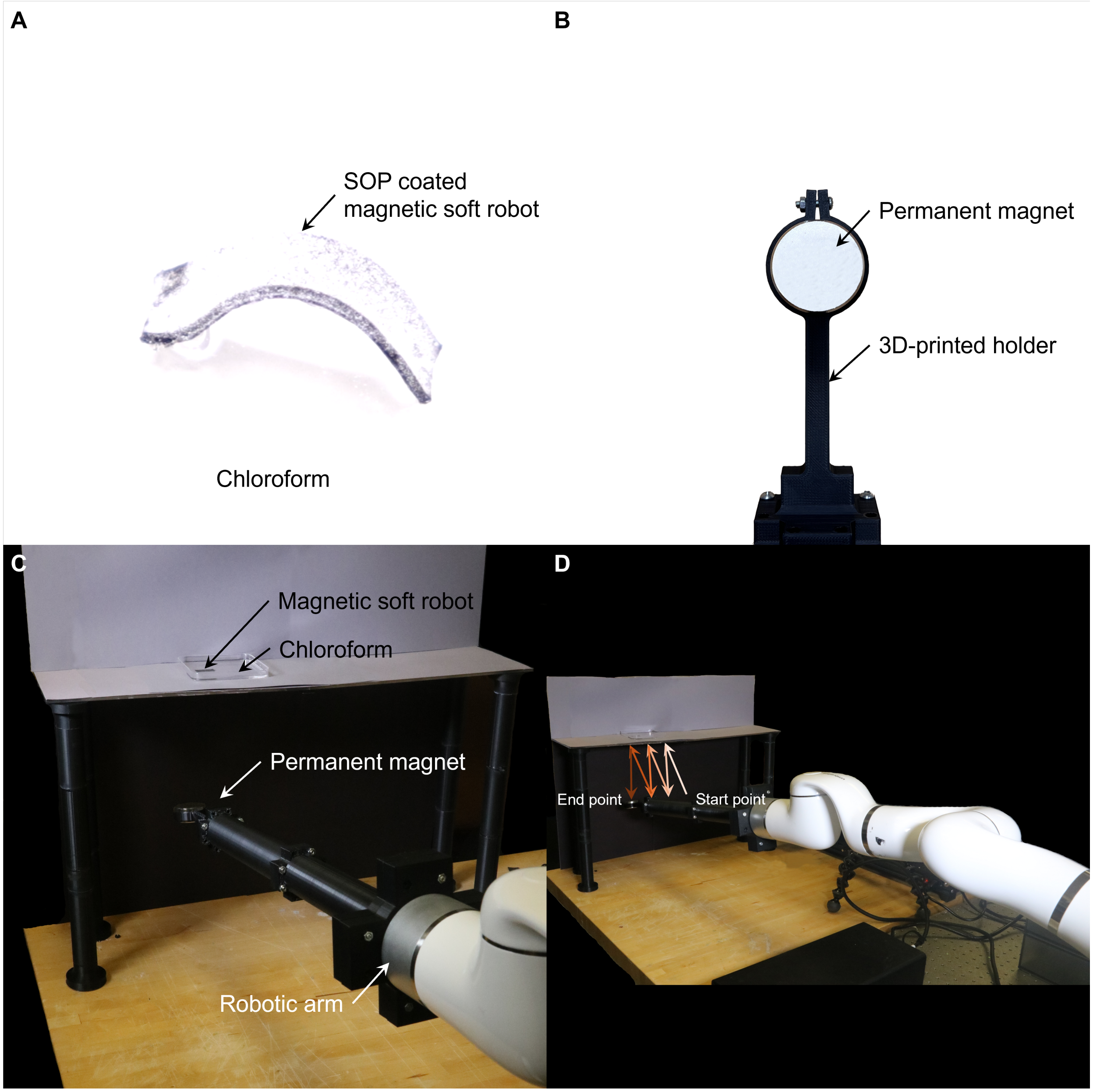

Figure S7. (**A**) Optical image of a superomniphobic coated magnetic soft robot submerged in chloroform. (**B**) Optical image showing the permanent magnet mounted on a robotic arm via a 3D-printed holder. (**C**) Experimental setup of the magnetic soft robot using a robotic arm. (**D**) Programmed trajectory of the magnet controlled by the robotic arm to manipulate the soft robot.

Supplementary movies legends

Movie S1. Droplet bouncing behaviors on superomniphobic silicone sheets (0% strain).

Movie S2. Droplet bouncing behaviors on superomniphobic silicone sheets (100% strain).

Movie S3. Chemical shielding performance of superomniphobic uncoated and coated ballons.

Movie S4. Chemical shielding performance of superomniphobic coated soft gripper.

Movie S5. Chemical shielding performance of superomniphobic coated fish-inspired soft swimmer.

Movie S6. Chemical shielding performance of superomniphobic coated jellyfish-inspired soft swimmer.

Movie S7. Chemical shielding performance of superomniphobic uncoated and coated LCE ribbons.

Movie S8. Chemical shielding performance of superomniphobic uncoated and coated magnetic crawlers.
